# Nobiletin Inhibits Inflammatory Reaction in Interleukin-1β-Stimulated Human Periodontal Ligament Cells

**DOI:** 10.3390/pharmaceutics13050667

**Published:** 2021-05-07

**Authors:** Yoshitaka Hosokawa, Ikuko Hosokawa, Kazumi Ozaki, Takashi Matsuo

**Affiliations:** 1 Department of Conservative Dentistry, Institute of Biomedical Sciences, Tokushima University Graduate School, Tokushima 770-8504, Japan; ihosokawa@tokushima-u.ac.jp (I.H.); matsuo.dent@tokushima-u.ac.jp (T.M.); 2 Department of Oral Health Care Promotion, Institute of Biomedical Sciences, Tokushima University Graduate School, Tokushima 770-8504, Japan; ozaki@tokushima-u.ac.jp

**Keywords:** nobiletin, anti-inflammatory effect, periodontal ligament cells

## Abstract

The immune response in periodontal lesions is involved in the progression of periodontal disease. Therefore, it is important to find a bioactive substance that has anti-inflammatory effects in periodontal lesions. This study aimed to examine if nobiletin, which is found in the peel of citrus fruits, could inhibit inflammatory responses in interleukin (IL)-1β-stimulated human periodontal ligament cells (HPDLCs). The release of cytokines (IL-6, IL-8, CXCL10, CCL20, and CCL2) and matrix metalloproteinases (MMP-1 and MMP-3) was assessed by ELISA. The expression of cell adhesion molecules (ICAM-1and VCAM-1) and the activation of signal transduction pathways (nuclear factor (NF)-κB, mitogen-activated protein kinases (MAPKs) and protein kinase B (Akt)) in HPDLCs were detected by Western blot analysis. Our experiments revealed that nobiletin decreased the expression of inflammatory cytokines, cell adhesion molecules, and MMPs in IL-1β-stimulated HPDLCs. Moreover, we revealed that nobiletin treatment could suppress the activation of the NF-κB, MAPKs, and Akt pathways. These findings indicate that nobiletin could inhibit inflammatory reactions in IL-1β-stimulated HPDLCs by inhibiting multiple signal transduction pathways, including NF-κB, MAPKs, and Akt.

## 1. Introduction

Periodontal disease is a chronic inflammatory disease caused by periodontal pathogens. The progression of periodontal disease induces the destruction of periodontium, including connective tissues and alveolar bone, and finally, teeth might be lost. The excessive immune reaction is involved in the initiation and advance of periodontal disease [1]. Leukocytes in periodontal lesions can produce too many inflammatory mediators, including cytokines [1] or matrix metalloproteinases (MMPs) [2]. Chemokines are related to the infiltration of leukocytes, and cell adhesion molecules could activate leukocytes in periodontal lesions [3]. Therefore, the reduction in chemokines, cell adhesion molecules, and MMP expression are important to prevent the progression of periodontal disease.

Nobiletin is a flavonoid that is isolated from the skin of citrus fruits, including tangerine [4]. It has been reported that nobiletin has various biological activities such as anticancer [5], antioxidant [6], antivirus [7], and anti-inflammatory effects [8,9]. Previous reports showed that nobiletin suppressed cell viability in prostate cancer cells [5]. Nobiletin could inhibit proinflammatory cytokine production in lipopolysaccharide (LPS)-stimulated RAW 264.7 cells [8]. Moreover, nobiletin inhibits phenylephrine-induced contractions of endothelium-denuded rat aorta by increasing cGMP levels via guanylate cyclase activation [9]. Furthermore, we found a report that revealed that nobiletin could inhibit LPS-induced bone resorption in a mouse experimental model for periodontal disease [10]. However, the mechanism of this phenomenon is not clear, and there are no reports that have examined the anti-inflammatory effect of nobiletin on periodontal resident cells.

This study aimed to examine the anti-inflammatory effects of nobiletin on inflammatory cytokines, cell adhesion molecules, and MMPs in interleukin (IL)-1β-stimulated human periodontal ligament cells (HPDLCs), which are cells that make up the majority in periodontal tissues and produce inflammatory mediators. Moreover, we examined if nobiletin can influence the activation status of mitogen-activated protein kinase (MAPK), nuclear factor (NF)-κB, and protein kinase B (Akt), which are activated by IL-1β stimulation, in HPDLCs.

## 2. Materials and Methods

### 2.1. Reagents

Nobiletin was purchased from Cayman Chemical (Ann Arbor, MI, USA). Recombinant human IL-1β was obtained from Peprotech (Rocky Hill, NJ, USA). Antibodies against intercellular adhesion molecule (ICAM)-1 and vascular cell adhesion molecule (VCAM)-1 were purchased from BioLegend (San Diago, CA, USA). Antibodies against phospho-p38 MAPK, phospho-extracellular signal-regulated kinase (ERK), phospho-c-Jun N-terminal kinase (JNK), phospho-IkappaB kinase (IKK)-α/β, phospho-NF-κB p65, phospho-Akt, p38 MAPK, ERK, JNK, IKK-α, NF-κB p65, Akt, and glyceraldehyde-3-phosphate dehydrogenase (GAPDH) were purchased from Cell Signaling Technology (Danvers, MA, USA). Enzyme-linked immunosorbent assay (ELISA) kits of IL-6, IL-8, CXCL10, CCL2, CCL20, MMP-1, MMP-3, and TIMP-1 were purchased from R&D Systems (Minneapolis, MN, USA).

### 2.2. Cell Culture

HPDLCs (Material Number: CC-7049, male, 16 years old) were obtained from Lonza Japan (Tokyo, Japan) and cultured in Dulbecco’s modified Eagle’s medium (Life Technology, Carlsbad, CA, USA) containing 10% fetal bovine serum (Sigma-Aldrich, St. Louis, MO, USA), 100 μg/mL streptomycin and 100 U/mL of penicillin (Life Technologies) at 37 °C in a humidified atmosphere of 5% CO_2_. The cells were used between passage numbers 5 and 10.

### 2.3. The Release of Cytokines and MMPs from HPDLCs

HPDLCs were seeded in a 24-well culture plate. The cells were incubated with different concentrations of nobiletin (12.5, 25, 50, or 100 μM) for 1 h and then stimulated with IL-1β (1 ng/mL) for 24 h. Next, the concentrations of IL-6, IL-8, CXCL10, CCL2, CCL20, MMP-1, MMP-3, and TIMP-1 in cell culture medium were measured using ELISA kits according to the manufacturer’s instructions.

### 2.4. Protein Extraction and Western Blot Analysis

HPDLCs were pretreated with nobiletin (25, 50, or 100 μM) for 1 h, and the HPDLCs were stimulated with IL-1β (1 ng/mL) for 15, 30, 60 min (analysis for the activation of signal transduction pathway) or 24 h (analysis for the adherent molecules expression). HPDLCs were washed twice with cold phosphate-buffered saline and collected using cold cell lysis buffer (Cell Signaling Technology: 20 mM Tris-HCl (pH 7.5), 150 mM NaCl, 1 mM Na_2_EDTA, 1 mM EGTA, 1% Triton, 2.5 mM sodium pyrophosphate, 1 mM beta-glycerophosphate, 1 mM Na_3_VO_4_, 1 µg/mL leupeptin) with protease inhibitor cocktail (Sigma-Aldrich). After cell lysates were centrifuged at 12,000× *g* for 10 min at 4 °C, the supernatants were collected. Total protein contents in cell lysates were measured by a bicinchoninic acid protein assay kit (Sigma-Aldrich). The same amount of total proteins were separated on sodium dodecyl sulfate-polyacrylamide gel electrophoresis (SDS-PAGE) and transferred to a polyvinylidene difluoride (PVDF) membrane (Millipore, Bredford, UK). Nonspecific protein bindings were blocked using 1% skim milk for 1 h at room temperature and the membrane was incubated with the specific primary antibodies at 4 °C for overnight: ICAM-1, VCAM-1, phospho-p38 MAPK, phospho-ERK, phospho- JNK, phospho-IKK-α β, phospho-NF-κB p65, p38 MAPK, ERK, JNK, IKK-α, or NF-κB p65. The GAPDH antibody was used to confirm equal protein loading. Horseradish Peroxidase-conjugated secondary antibodies (Sigma) bound to the primary antibodies were detected using the Enhanced Chemi Luminescence (ECL) Plus Western blotting detection system (GE Healthcare, Uppsala, Sweden) according to the manufacturer’s instructions. The experiments were repeated three times. The quantitation of the chemiluminescent signal was analyzed using ImageJ (version 1.52p: NIH, Bethesda, MD, USA).

### 2.5. Statistical Analysis

**Figure 1:**
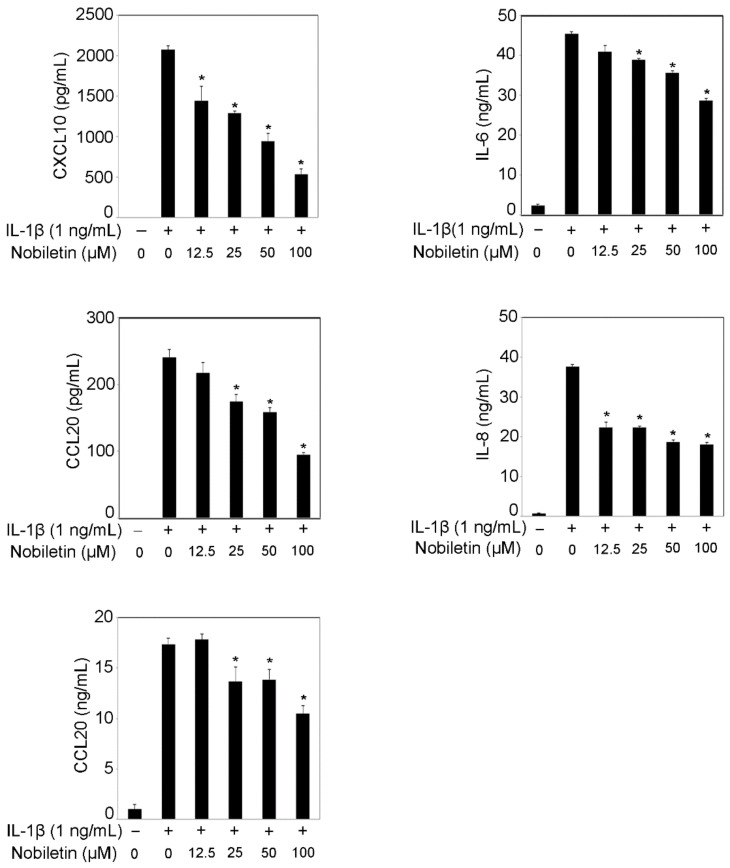
Effects of nobiletin on CXCL10, CCL2, CCL20, IL-6, and IL-8 production in IL-1β-stimulated HPDLCs. HPDLCs were pretreated with nobiletin (12.5, 25, 50, or 100 μM) for 1 h before being stimulated with IL-1β (1 ng/mL) for 24 h. After stimulation, the culture supernatants were collected and the levels of CXCL10, CCL2, CCL20, IL-6, and IL-8 were measured using ELISA kits. Data are expressed as the mean and ±standard deviation (SD) and are representative of three independent experiments. * *p* < 0.01 significantly different from the concentration of the IL-1β-treated HPDLCs that were not treated with nobiletin.

**Figure 2:**
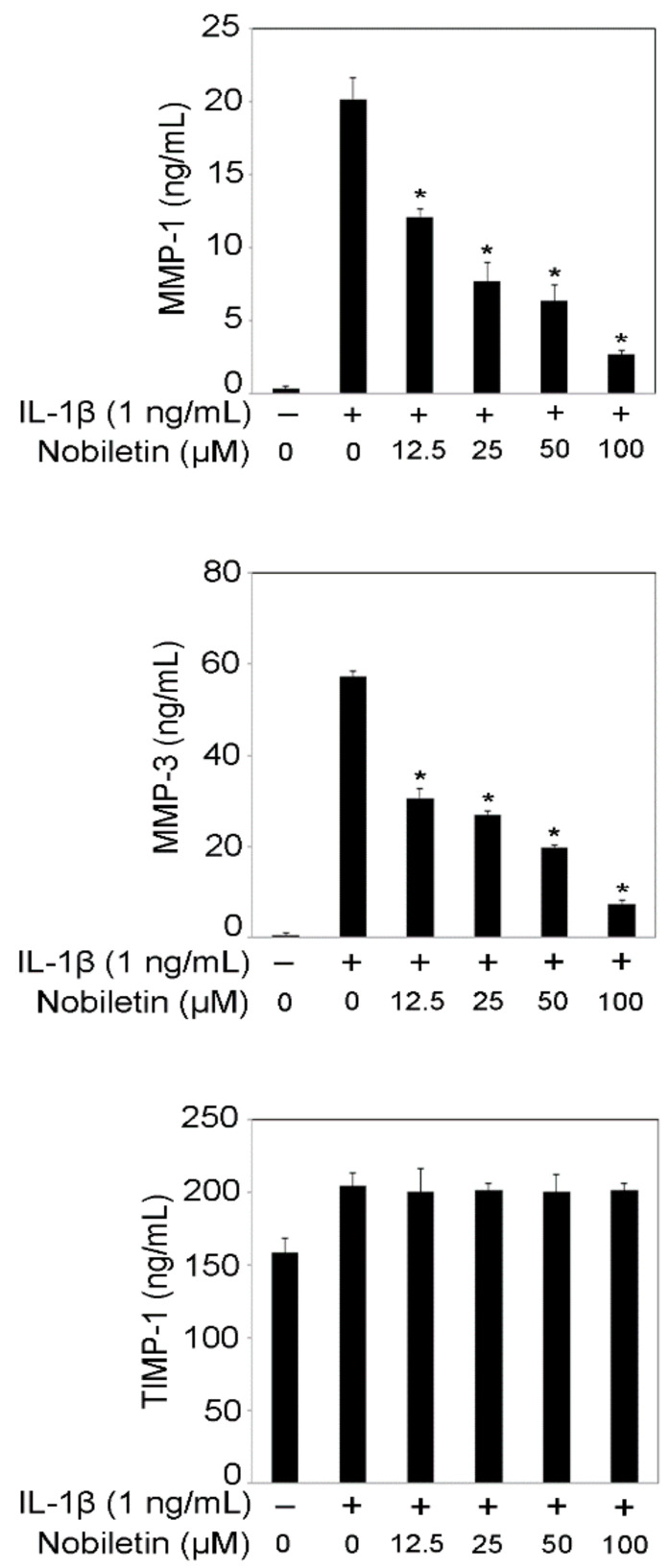
Effects of nobiletin on MMP-1, MMP-3, and TIMP-1 production in IL-1β-stimulated HPDLCs. HPDLCs were pretreated with nobiletin (12.5, 25, 50, or 100 μM) for 1 h, before being stimulated with IL-1β (1 ng/mL) for 24 h. After stimulation, the supernatants were collected and the concentrations of MMP-1, MMP-3, and TIMP-1 were measured using ELISA kits. Data are expressed as the mean and ± SD of one representative experiment and are representative of three independent experiments. * *p* < 0.01 is significantly different from the concentration of the IL-1β-treated HPDLCs that were not treated with nobiletin.

Statistical significance was analyzed using one-way ANOVA. *p*-values of < 0.05 were considered significant in the analyses shown in Figure 1 and Figure 2.

## 3. Results

### 3.1. Nobiletin Could Inhibit Inflammatory Cytokine Production in IL-1β-Stimulated HPDLCs

We previously reported that IL-1β treatment enhanced inflammatory cytokine production, including IL-6, IL-8, CXCL10, CCL2, or CCL20, in HPDLCs [11,12]. Excessive inflammatory cytokine expressions in periodontal lesions are related to the pathogenesis of periodontal disease [1]. It is certain that IL-8, CXCL10, CCL2, and CCL20 regulate neutrophils, Th1 cells, macrophages, and Th17 cells accumulation, respectively [13]. IL-6 could enhance osteoclast differentiation in inflammatory lesions [14]. Therefore, it is important to examine if nobiletin can decrease chemokine (IL-8, CXCL10, CCL2, and CCL20) and IL-6 production. Figure 1 clearly shows that nobiletin significantly inhibits CXCL10, CCL2, CCL20, IL-6, and IL-8 production in IL-1β-stimulated HPDLCs in a dose-dependent fashion.

### 3.2. Nobiletin Inhibited MMP-1 and MMP-3 Production in IL-1β-Stimulated HPDLCs

MMPs that could degrade the extracellular matrix are involved in the destruction of the connective tissues in periodontal lesions [2]. Periodontal tissues include type 1 collagen, and MMP-1 could progress periodontal soft tissues destruction because MMP-1 can degrade type 1 collagen. MMP-3 is also involved in soft tissue destruction as it can activate pro-MMP-1 [2]. Therefore, inhibition of MMP-1 and MMP-3 production leads to the treatment of periodontal disease. Figure 2 shows that nobiletin treatment decreased MMP-1 and MMP-3 production HPDLCs stimulated with IL-1β in a dose-dependent manner. Nobiletin treatment did not change TIMP-1 production in IL-1β-stimulated HPDLCs. Thus, MMP-1 and MMP-3 activity is decreased by nobiletin treatment.

### 3.3. Nobiletin Decreased ICAM-1 and VCAM-1 Expression in IL-1β-Stimulated HPDLCs

**Figure 3:**
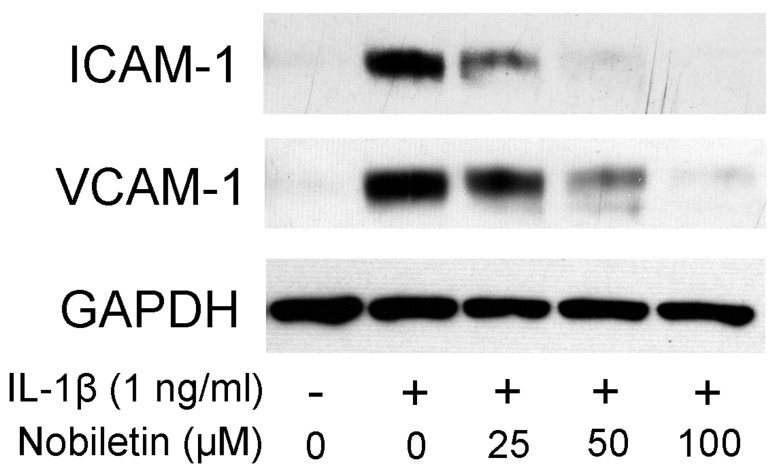
Effects of nobiletin on ICAM-1 and VCAM-1 expression in IL-1β-stimulated HPDLCs. HPDLCs were pretreated with nobiletin (25, 50, or 100 μM) for 1 h before being stimulated with IL-1β (1 ng/mL). The lysates were collected after 24 h. The expressions of ICAM-1 and VCAM-1 were examined by Western blot analysis. Each photograph is representative of the results of 3 separate experiments.

It was reported that HPDLCs could express ICAM-1 and VCAM-1 and that adherent molecules are involved in the retention and activation of leucocytes in inflammatory lesions [15]. Therefore, we examined the effect of nobiletin on ICAM-1 and VCAM-1 expression in IL-1β-treated HPDLCs. Figure 3 shows that IL-1β stimulation enhanced ICAM-1 and VCAM-1 expression and the levels of ICAM-1 and VCAM-1 expression were decreased by nobiletin treatment.

### 3.4. Nobiletin Decreased the Level of p38 MAPK, ERK, and JNK Phosphorylation in IL-1β-Stimulated HPDLCs

**Figure 4:**
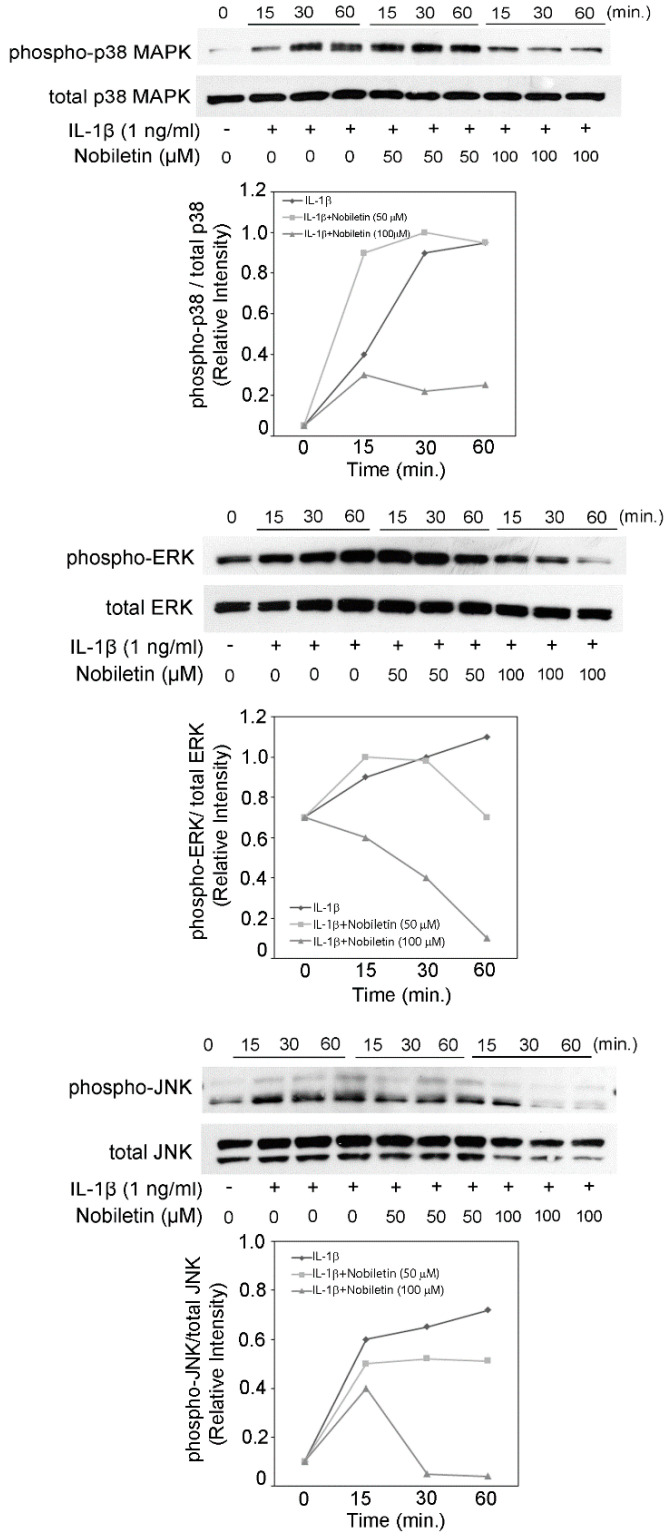
The effects of nobiletin on the intracellular phosphorylation of p38 MAPK, ERK, and JNK in IL-1β-stimulated HPDLCs. HPDLCs were treated with nobiletin (0, 50, or 100 μM) for 24 h, followed by stimulation with or without IL-1β (1 ng/mL) for 15, 30, or 60 min. Western blot analysis was conducted to assess the expression of phospho-p38 MAPK, p38 MAPK, phospho-ERK, ERK, phospho-JNK, JNK and, GAPDH. Each photograph is representative of data of 3 separate experiments.

Our previous reports described the IL-1β stimulation could enhance the phosphorylation level of p38 MAPK, ERK, and JNK in HPDLCs, and inhibitors of MAPKs could inhibit inflammatory mediator production [12,16]. We previously reported that IL-6, IL-8, CCL2, CCL20, CXCL10, MMP-1, and MMP-3 production in HPDLCs were decreased by a p38 MAPK inhibitor, an ERK inhibitor, or a JNK inhibitor [11,12,16,17]. Lee et al. reported that p38 MAPK and ERK activation is involved in ICAM-1 expression in HPDLCs [18], and it is reported that VCAM-1 expression in HPDLCs was inhibited by an ERK inhibitor [19]. Therefore, we examined the effect of nobiletin on MAPK activation in this study. Figure 4 shows that 100 μM nobiletin treatment could decrease the phosphorylation level of p38 MAPK and ERK in IL-1β-stimulated HPDLCs, though 50 μM nobiletin caused no inhibition. The phosphorylation level of JNK was inhibited by 50 and 100 μM nobiletin treatments.

### 3.5. Nobiletin Could Inhibit Akt Phosphorylation in IL-1β-Stimulated HPDLCs

**Figure 5:**
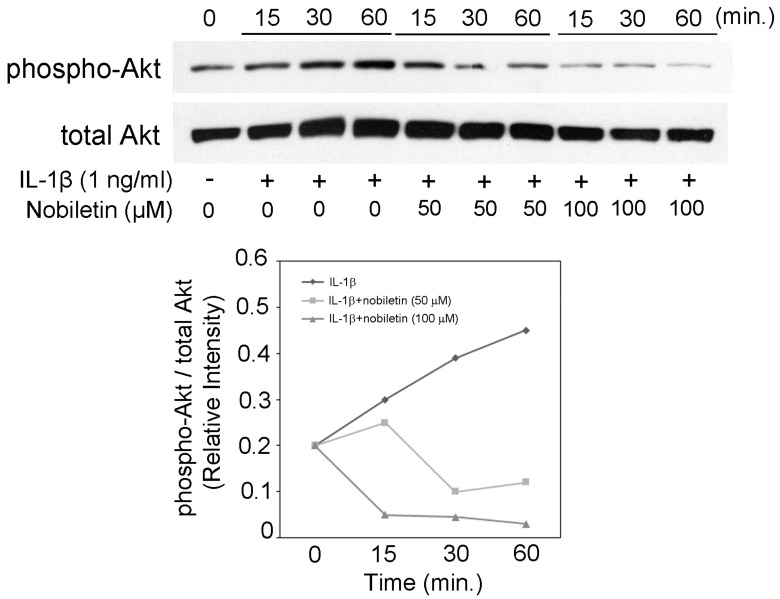
The effects of nobiletin on the intracellular phosphorylation of Akt in IL-1β-stimulated HPDLCs. HPDLCs were treated with nobiletin (0, 50, or 100 μM) for 24 h, followed by stimulation with or without IL-1β (1 ng/mL) for 15, 30, or 60 min. Western blot analysis was conducted to assess the expression of phospho-Akt, Akt, and GAPDH. Each photograph is representative of the results of 3 separate experiments.

Akt is a key signal transduction molecule that is related to the production of various inflammatory mediators. Previous manuscripts including ours show that an Akt inhibitor suppressed IL-6, IL-8, CCL20, MMP-1, and MMP-3 production in HPDLCs [20,21,22], though there are no reports that examined the effect of an Akt inhibitor on CCL2, CXCL10, ICAM-1, and VCAM-1 expression. Therefore, we would like to investigate the influence of nobiletin on the level of Akt phosphorylation. Our results indicate that 50 or 100 μM nobiletin suppressed the phosphorylation level of Akt in IL-1β-stimulated HPDLCs (Figure 5). This result means that the inhibitory effect of nobiletin on Akt pathway activation might be involved in the decrease in IL-6, IL-8, CCL20, MMP-1, and MMP-3.

### 3.6. Nobiletin Could Inhibit NF-κB Activation in IL-1β-Stimulated HPDLCs

**Figure 6:**
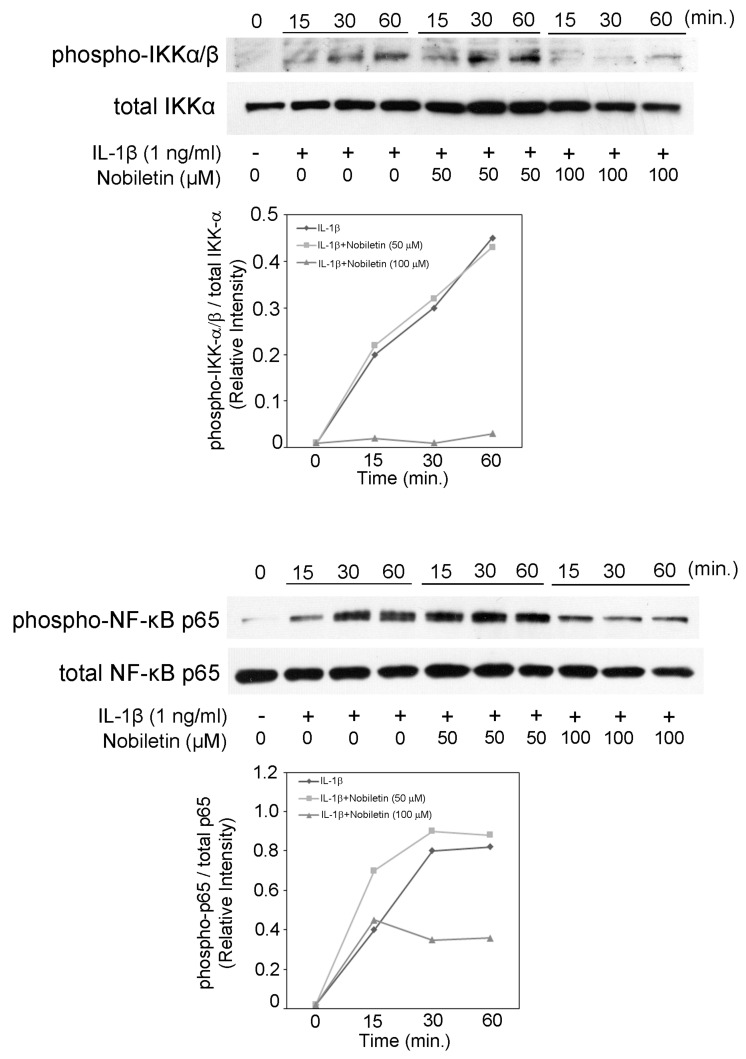
The effects of nobiletin on the intracellular phosphorylation of IKK-α/β and NF-κB p65 in IL-1β-stimulated HPDLCs. HPDLCs were treated with nobiletin (0, 50, or 100 μM) for 24 h, followed by stimulation with or without IL-1β (1 ng/mL) for 15, 30, or 60 min. Western blot analysis was conducted to assess the expression of phospho-IKK-α/β, phospho-NF-κB p65, IKK-α, NF-κB p65, and GAPDH. Each photograph is representative of the results of 3 separate experiments.

It is well-known and important that NF-κB pathway activation is involved in inflammatory mediator production. Previous manuscripts including ours reported that an NF-κB signal pathway is positively involved in the expressions of inflammatory cytokines (IL-6, IL-8), chemokines (CCL2, CCL20, CXCL10), MMPs (MMP-1, MMP-3), and adherent molecules (ICAM-1, VCAM-1) in HPDLCs [11,12,16,17,18,19,20]. In this study, we examined the effect of nobiletin on IKK-α β and NF-κB p65 phosphorylation in HPDLCs. Figure 6 shows that 100 μM nobiletin suppressed the level of IKK-α β and NF-κB p65 phosphorylation in IL-1β-treated HPDLCs.

## 4. Discussion

The excessive immune reaction is involved in the progression of periodontal disease [1]. It is important to find a substance that could decrease the inflammatory response in periodontal lesions. In this study, we report that nobiletin could inhibit inflammatory cytokine, MMP, and cell adhesion molecule expression in HPDLCs. High amounts of inflammatory cytokines and chemokines can activate osteoclasts in periodontal lesions and induce alveolar bone loss [23]. MMPs could degrade extracellular matrix in periodontal disease tissues and are related to soft tissue destruction [2]. Cell adhesion molecules on HPDLCs are involved in the retention and activation of leukocytes in periodontal lesions [24]. Nobiletin may dramatically suppress the progression of periodontal disease to decrease the three kinds of inflammatory mediators concerned. Further study using the disease model of the animal is necessary.

We show that nobiletin treatment could inhibit the activation of p38 MAPK, ERK, and JNK in IL-1β-stimulated HPDLCs. Kim et al. showed that nobiletin could inhibit p38 MAPK phosphorylation in PMA-stimulated human dermal fibroblasts, though nobiletin did not modulate ERK and JNK phosphorylation [25]. Zhou et al. reported that nobiletin decreased the level of JNK phosphorylation in angiotensin Ⅱ-treated vascular smooth muscle cells, though the levels of p38 MAPK and ERK did not change due to nobiletin treatment [26]. Judging from our report and previous reports, the effects of nobiletin on MAPK activation may vary according to the kinds of cells or the kind of stimulation.

We describe that nobiletin could suppress the activation of the NF-κB pathway in HPDLCs stimulated with IL-1β. Lin et al. recently reported that nobiletin treatment could inhibit IκB-α phosphorylation of IκB-α and NF-κB p65 in IL-1β-stimulated mouse chondrocytes [27]. Li et al. reported that nobiletin could decrease the levels of NF-κB p65 and IκB-α phosphorylation in LPS-treated A549 cells (human alveolar adenocarcinoma cell line) [28]. We think that, as a common property, nobiletin controls the activation of the NF-κB pathway. It is thought that the suppression of the NF-κB pathway is very important in explaining the anti-inflammatory effect of nobiletin.

We revealed that nobiletin could inhibit the level of Akt phosphorylation in IL-1β-stimulated HPDLCs. We found some reports that investigated the effect of nobiletin on Akt phosphorylation. Nobiletin suppressed IL-1β-induced proinflammatory mediators, including prostaglandin E2, nitric oxide, cyclooxygenase-2, inducible nitric oxide synthase, tumor necrosis factor-a, and IL-6 by inhibiting Akt and NF-κB activation in human osteoarthritis chondrocytes [29]. Qi et al. reported that pretreatment of nobiletin diminished the secretion of proinflammatory cytokines in lipopolysaccharide-stimulated BV-2 microglia cells by modulating MAPK, Akt, and NF-κB pathways [30]. Our report and previous reports explain that the inhibitory effect of nobiletin on Akt pathway activation is related to the anti-inflammatory effects of nobiletin.

## 5. Conclusions

This study is the first to demonstrate the anti-inflammatory activity of nobiletin in HPDLCs. Nobiletin significantly inhibited the IL-1β-induced inflammatory response and suppressed the MAPK, NF-κB, and Akt signaling pathways. Thus, nobiletin may be a potential agent in the treatment of periodontal disease.

## Data Availability

The data presented in this article are openly available.
